# Contrasting subtropical PV intrusion frequency and their impact on tropospheric Ozone distribution over Pacific Ocean in El-Niño and La-Niña conditions

**DOI:** 10.1038/s41598-017-12278-7

**Published:** 2017-09-20

**Authors:** Debashis Nath, Wen Chen, Hans-F. Graf, Xiaoqiang Lan, Hainan Gong

**Affiliations:** 1 Center for Monsoon System Research, Institute of Atmospheric Physics, Chinese Academy of Sciences, Beijing, 100190 China; 2 Center for Atmospheric Science, University of Cambridge, Cambridge, UK

## Abstract

Upper tropospheric equatorial westerly ducts over the Pacific Ocean are the preferred location for Rossby wave breaking events during boreal winter and spring. These subtropical wave breaking events lead to the intrusion of high PV (potential vorticity) air along the extra-tropical tropopause and transport ozone rich dry stratospheric air into the tropics. The intrusion frequency has strong interannual variability due to ENSO (El-Niño/Southern Oscillation), with more events under La-Niña and less under El-Niño conditions. This may result from stronger equatorial westerly ducts and subtropical jets during La-Niña and weaker during El-Niño. It was previously suggested that the interannual variability of the tropospheric ozone distribution over the central-eastern Pacific Ocean is mainly driven by convective activity related to ENSO and that the barotropic nature of the subtropical intrusions restricts the tracers within the UT. However, our analysis shows that tropospheric ozone concentration and subtropical intrusions account ~65% of the co- variability (below 5 km) in the outer tropical (10–25°N) central Pacific Ocean, particularly during La-Niña conditions. Additionally, we find a two-fold increase and westward shift in the intrusion frequency over the Pacific Ocean, due to the climate regime shift in SST pattern during 1997/98.

## Introduction

The extra-tropical-tropical interactions and the equatorward transport of stratospheric dry air have significant impact on the tropical dynamics, particularly in the central and eastern Pacific^1^. During boreal winter and spring, upper tropospheric (UT) equatorial westerly ducts over the Pacific and the Atlantic oceans are the preferred location for stratosphere-troposphere exchanges^1^. Cross equatorial Rossby wave propagation is possible through these westerlies, and breaking may occur if the wave amplitude is sufficiently large^2^. This may result in reduced meridional wave propagation and mixing within the equatorial regions^3,4^. These wave breaking events can be seen in maps of PV as tongues of high PV air intruding into the tropics along the extra-tropical tropopause^5^. The disturbances are compressed zonally and are amplified meridionally as they enter the weak zonal wind regime, north of the westerly duct^1^. These intrusions transport stratospheric air deep into the tropics, and affect the UT distribution of ozone, water vapor and other trace constituents^1^. They may contribute to the observed increase in ozone below the tropical tropopause. Their penetration into the tropics can be linked to the strength of convection and westerly wind over the tropical central and eastern Pacific. Waugh and Funatsu^6^ and Waugh^7^ analyzed the impact of Pacific intrusions on convection, tropospheric ozone distribution and subtropical humidity using outgoing long-wave radiation (OLR), Hilo ozonesonde and Microwave Limb Sounder (MLS) satellite measurements, respectively. However, their studies are restricted till the end of 20^th^ century. The interannual variability of the PV intrusions correlates strongly with the phase of El-Niño/Southern Oscillation (ENSO). There are fewer cases in the warm (EN) with weaker westerlies and more cases in the cold (LN) phase of ENSO when stronger westerlies occur in the equatorial UT. In the EN phase, the subtropical jet is stronger, whereas it is weaker in the LN phase. The related variation of the diffluence strength in the jet exit region further modulates the frequency of the PV intrusion events. Hence, the long term and interannual variability in UT zonal wind should have a significant impact on the PV intrusion frequency over the Pacific Ocean.

**Figure 1:**
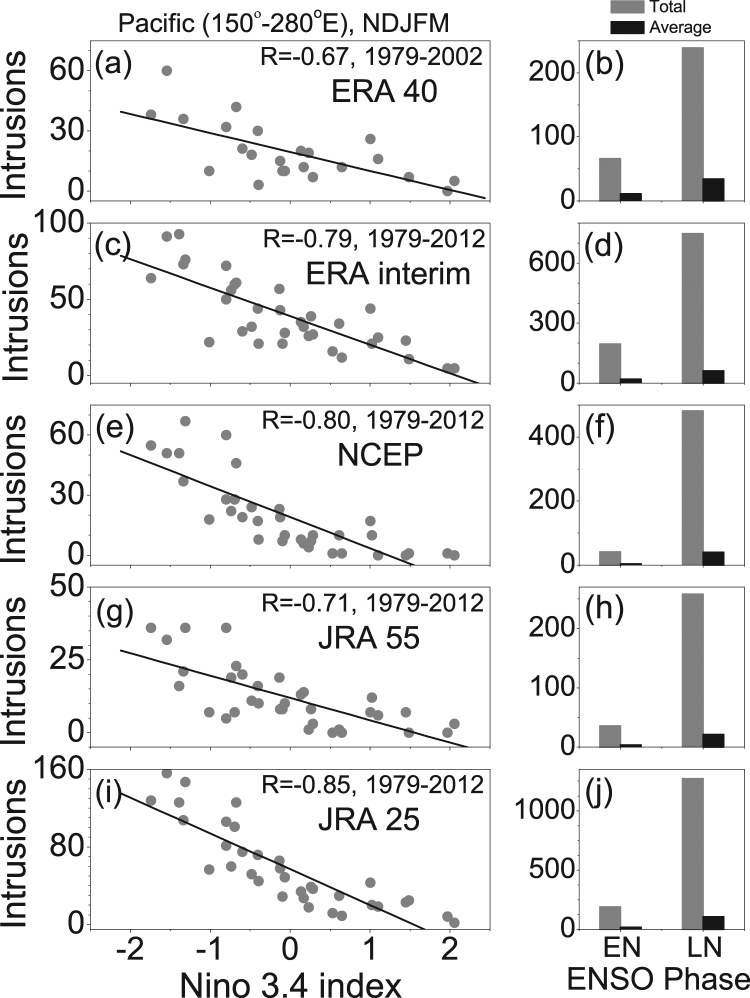
PV intrusion frequency during EN and LN. (**a**,**c**,**e**,**g**,**i**) Represent correlation coefficient between NDJFM mean Nino 3.4 index and Pacific PV (150°–280°E) intrusion events for ERA 40 (1979–2002), ERA interim (1979–2012), NCEP (1979–2012), JRA 55 (1979–2012) and JRA 25 (1979–2012), respectively. The significant (>95%) correlation coefficient (R) value is written within each subplots. (**b**,**d**,**f**,**h**,**j**) Represent the total (gray bar) and average (black bar) number of Pacific intrusions in the El-Nino (EN) and La-Nina (LN) years for the respective datasets mentioned above.

Drawn from multiple reanalysis datasets (ECMWF, NCEP, Japanese Reanalysis, MERRA, etc.), an increasing trend and westward shift in the number of PV intrusion events over the Pacific Ocean are observed^8^. Nath *et al*.^8^ linked this increased frequency to the long-term trend in upper tropospheric equatorial westerly wind and subtropical jets during boreal winter to spring months, which may result from anomalous warming over the western Pacific warm pool and cooling over the tropical eastern Pacific Ocean. Apart from the interdecadal trend, it is interesting to investigate the interannual variability in PV intrusion frequency, especially in relation with the Pacific SST. A two-fold increase in intrusion frequency is evident since the end of the 1990’s (right panel of Fig. 1, Nath *et al*.^8^). This time period is consistent with a climate regime shift (CRS) in the Pacific Ocean^9^ around 1997/98, after which a LN like pattern dominates the SST pattern. Therefore, we also investigate the variability in PV intrusion frequency in relation with the SST anomalies over the Pacific Ocean during the pre-1998 and post-1998 periods. In the present manuscript, we employ the same methodologies and datasets as in Nath *et al*.^8^ to characterize the PV intrusion events, now separately for El-Niño (EN) and La-Niña (LN) conditions.

As mentioned above, these intrusions bring dry and ozone rich air of stratospheric origin deep into the tropical troposphere. Despite the importance of PV intrusion (PVI) events on the tropical and subtropical dynamics, only few studies highlight the climatology of intrusions related to stratosphere-troposphere exchange (STE) and tropospheric ozone distribution. Using the ECMWF reanalysis dataset, Škerlak *et al*.^10^ compiled a global climatology of STE from 1979 to 2011. Ozone fluxes are calculated across the tropopause, across pressure surfaces in the troposphere, and across the top of the planetary boundary layer. This climatology also provides a quantification of the geographical distribution of STE and the preferred transport pathways, as well as it allows insight into the temporal evolution of STE during the analysis period. The global hotspots for deep STE are found along the west coast of North America and over the Tibetan Plateau, especially in boreal winter and spring. A climatological analysis by Ryoo *et al*.^11^ clearly demonstrates the role of Rossby wave breaking and STE processes for U.S. west coast winter precipitation in the two phases of ENSO. Therefore, it is important to investigate the interannual and long-term variability in the STE processes in connection with the PV intrusion frequency and ozone transport in the tropics.

The subtropical intrusions bring dry and ozone rich air of stratospheric origin deep into the tropics (Nath *et al*.^8^). It is also accepted that interannual ozone variability in the tropical UT is mainly related to convection associated with El Niño/Southern Oscillation (Oman *et al*.^12^). The zonal mean stratospheric overturning circulation (Brewer-Dobson circulation (BDC); Butchart *et al*.^13^ organizes the transport of ozone rich air poleward and downward to the high and mid-latitudes, leading there to increased ozone concentrations. Moreover, a good part of ozone is also transported from the tropics to higher latitudes more rapidly by Rossby wave mixing in the lower stratosphere (Neu *et al*.^14^). In addition to these well described mechanisms, Nath *et al*.^8^, observed a long-term increasing trend in ozone flux from the mid latitudes to the northern hemispheric outer tropical central Pacific Ocean. The TOMS (total column) and AIRS (vertical profile) ozone concentration exhibits an increasing trend in the outer tropics and a decreasing trend in mid latitudes, which is in line with the trends in PV inducing equatorward transport and downward mixing of ozone from the midlatitude UT and lower stratosphere during PV intrusions. Hsu *et al*.^15^ used a westerly duct event in March 2001 to demonstrate the stratospheric ozone intrusion into the tropical eastern Pacific as observed by TOMS. However, the net influx associated with the event is much less than the anomalous amount seen in the total columnar ozone. They also reported that the STE fluxes in the warm phase are distinctly different from the cold phase of ENSO. Furthermore, the vertical extent of the ozone transport due to subtropical intrusions has not been examined in details, except few case studies by Waugh and Funatsu^6^ and Funatsu and Waugh^13^. They showed that the narrow tongues of high PV air with north-south orientation extend into the lower stratosphere (LS), but only few penetrate deeper into the tropical UT. The subtropical intrusions in general are fairly barotropic, remain in the UT and do not penetrate down to the lower troposphere (LT)^7^.

Recently, Neu *et al*.^14^ reported that the zonal mean stratospheric circulation driven by ENSO and Quasi-Biennial Oscillation (QBO) increases the ozone fluxes from the stratosphere to the troposphere by 2%, approximately half of the interannual variability. The poleward and downward transport of ozone gets enhanced during EN/easterly shear QBO and suppressed during LN/westerly shear QBO. During EN the decrease in UT ozone concentration is related to the enhanced convection over the central-eastern Pacific Ocean, while during LN suppressed convection leads to increased ozone concentration. This results in a negative (positive) zonal mean ozone anomaly during EN (LN). In the mid-latitudes, transport of ozone from the stratosphere to the troposphere is frequent near the subtropical jet (30°–40°N) and is one of the primary sources of tropospheric ozone. In addition to these well described mechanisms, we observe an increasing equatorward transport and downward mixing of ozone from the midlatitude UT and LS during PV intrusions under La Nina conditions. Several previous studies of tropopause folds relate ozone and PV near the tropopause, but they mainly focused on the mid-latitudes. Only few of the studies are dealing with ozone transport related to subtropical events^6,16–18^. Here, we focus on the climatology of PV intrusions, the variability of UT zonal wind, and its impact on tropospheric ozone concentration over the central Pacific Ocean in the two phases of ENSO. Different reanalysis datasets and satellite measurements are used to assess the impact of Rossby wave breaking on tropospheric ozone levels during EN and LN conditions. We also discuss the factors other than subtropical intrusions, e.g. tropical convection, stratospheric overturning circulation, vertical velocity and tropopause height, affecting the variability of tropospheric ozone fluxes in the troposphere. Finally, we highlight the interdecadal variability of SST, UT zonal wind, intrusion frequency and ozone transport in relation with the CRS over the Pacific Ocean.

## Data and Analysis

### Data used

Five reanalysis datasets, the European Centre for Medium–Range Weather Forecasts (ECMWF) (ERA40^19^ and ERA interim^20^), National Centers for Environmental Prediction’s (NCEP^21^), and Japanese Reanalysis (JRA55^22^ and JRA25^23^) are used to examine the climatology of PV intrusion from 1979 to 2012 for the winter months (November to March). Daily mean PV at 350 K isentropic surfaces (NCEP & JRA) or 200 hPa (ECMWF) are used to estimate the climatology of Rossby wave breaking events over the Pacific Ocean. Additionally, monthly mean zonal and meridional wind fields are used to calculate the basic and divergent flow fields. The monthly mean SST, SLP, and Niño 3.4 index data are available from the Met Office Hadley Centre and the NOAA Climate Prediction Center, respectively. In the present manuscript, winter mean for all the variables are computed from 1979 to 2012 (2001 for ERA 40). For vertical profiles of PV, Ozone mass mixing ratio and Ozone volume mixing ratio, we used ERA interim and satellite borne Atmospheric Infra–Red Sounder (AIRS), version 6, between 1979–2012 and 2003–2012, respectively.

The AIRS data are available in 360 × 180 × 24 (longitude × latitude × pressure) grids from the ground to 48 km height. The retrieval biases compared with global ozonesondes are less than 5%, both for the stratosphere and the troposphere, and the RMS differences are less than 20% in the LS and the troposphere^22^. Four different Total Ozone mapping Spectrometer (TOMS) instruments were operational at different periods during the 30 years of interest. In our analysis we use the total columnar ozone data (DU) from *Nimbus–7* (January 1983 to May 1993), *Meteor–3* (May 1993 to November 1994), *Earth Probe* (July 1996 to December 2004), and *OMI* (January 2005–December 2012). TOMS data between November 1994 and July 1996 are unavailable and the data gaps are filled by linear interpolation. For simplicity of the analysis, all the datasets are interpolated linearly to a 360 × 180 (longitude × latitude) grid.

### ENSO classification

We classified the warm (EN, 1982/83, 1986/87, 1987/88, 1991/92, 1994/95, 1997/98, 2002/03, 2004/05, 2006/07 and 2009/10) and cold (LN, 1983/84, 1984/85, 1988/89, 1995/96, 1998/99, 1999/00, 2000/01, 2005/06, 2007/08, 2008/09, 2010/11 and 2011/12) ENSO phases based on NDJFM mean Niño 3.4 index, with values greater and lesser than 0.5 (EN) and −0.5 (LN), respectively. In the composite analyses of different variables, we considered the mean of 10 warm and 12 cold events, respectively. For AIRS Ozone density, due to the shorter time period, we only have 4 cases with EN and 5 with LN conditions.

### PV intrusion

PV Intrusions are defined by identifying high values of PV (|PV| > 2 PVU, 1PVU = 10^−6^ Ks^2^/kg) at 10°N or 10°S. All such cases with 10° longitude or within 6 days are grouped as a single intrusion event. Small blobs (spread less than 10°) of high PV in the tropics are removed, to exclude the cases which are not immediately related with Rossby wave breaking^1^, e.g. a large undulation of PV without detachment of the PV filament from its source is considered no real breaking event. Pacific intrusions are characterized by the number of events within 150°–280°E. However, it is worth mentioning in this context that, in the literature there are arguments for the importance of depth and strength of STE rather than just frequency^10,24^. Therefore in the later stage of our manuscript, we also have analyzed the vertical extent of PV intrusion in the two phases of ENSO.

### EOF analysis

Empirical Orthogonal function^25^ (EOF) analysis is used to obtain the dominant variability modes of seasonal mean UT (200 hPa) zonal wind for the five reanalysis datasets. EOFs are orthogonal spatial patterns that can be thought of as empirically derived basic functions. The low–order EOFs can be interpreted as natural modes of variation of the observed system. However, it is worth mentioning in this context that the EOF analyses are purely statistical results and their physical meaning needs to be checked and proved independently. The coefficients that are obtained by projecting the observed field onto the EOFs, called principal components (PCs), are uncorrelated and represent the temporal variability of the field. The PC1 (first PC) time series is standardized by dividing the mean removed trend with the standard deviation. The variance explained by the PC1 (dominant) is expressed in percentage. We tested the significant differences among the modes in terms of explained variances and PC1 appears to be the dominant one. The results from the EOF analysis are confirmed by addressing the changes in zonal wind at 200 hPa and from multiple reanalysis datasets.

### Divergent wind

The divergent wind is calculated splitting the horizontal wind velocity into non–divergent (rotational) and divergent components^26,27^. The rotational components of the horizontal winds make no contribution to atmospheric divergence associated with vertical motion.

### Plumb Flux

The concept is introduced to analyze the wave propagation from the troposphere to the stratosphere in the three-dimensional space^28^. In the log-pressure coordinates, the wave activity flux *F*_*s*_ can be represented as follows:

$${F}_{s}=pcos\theta (\begin{array}{c}\begin{array}{c}\frac{1}{2{r}^{2}co{s}^{2}\theta }[{(\frac{\partial \psi ^{\prime} }{\partial \lambda })}^{2}-\psi ^{\prime} \frac{{\partial }^{2}\psi ^{\prime} }{\partial {\lambda }^{2}}]\\ \frac{1}{2{r}^{2}cos\theta }[\frac{\partial \psi ^{\prime} }{\partial \lambda }\,\frac{\partial \psi ^{\prime} }{\partial \theta }\,\mbox{--}\,\psi ^{\prime} \frac{{\partial }^{2}}{\partial \lambda }\frac{\psi ^{\prime} }{\partial \theta }]\end{array}\\ \frac{2{\Omega }^{2}si{n}^{2}\theta }{{N}^{2}rcos\theta }[\frac{\partial \psi ^{\prime} }{\partial \lambda }\frac{\partial \psi ^{\prime} }{\partial z}-\psi ^{\prime} \frac{{\partial }^{2}}{\partial \lambda }\frac{\psi ^{\prime} }{\partial z}]\end{array})$$

where $\psi ,\lambda ,\,\Omega ,z,{\rm{and}}\,p$ are the streamfunction, longitude, Earth’s rotation rate, altitude, and pressure/1000 hPa, respectively. The primes in the equation represent the perturbation fields.

### PV tropopause

The dynamical tropopause height is derived from ERA interim reanalysis data with PV = 2PVU unit^29^.

## Results

### Variability of PV intrusion events in two phases of ENSO

ENSO has significant impact on lateral transport of tracers into the tropics by means of intrusion and the interannual variability of Pacific PVI events correlates strongly with the Niño 3.4 index. Previous studies reported that the impact of ENSO on the Pacific UT field^30,31^ is maximum during boreal winter and spring months (November-March), much larger than in any other season of the year. Figure 1(a,c,e,g and i) display the correlation coefficients between Niño 3.4 and Pacific PVI events (sum of intrusions at 10°N and 10°S) during the NDJFM months for ERA 40, ERA interim, NCEP, JRA 55 and JRA 25, which is −0.67, −0.79, −0.8, −0.71 and −0.85, respectively. The results also indicate fewer intrusions during the warm and more during the cold phase of ENSO. Despite some obvious differences in the number of PVI events among different datasets, the total and average number of events during LN is much higher than under EN conditions. The result is consistent and unequivocal in all five datasets (Fig. 1(b,d,f,h and j)).

### Variability in UT zonal wind and tropical circulations in two phases of ENSO

**Figure 2:**
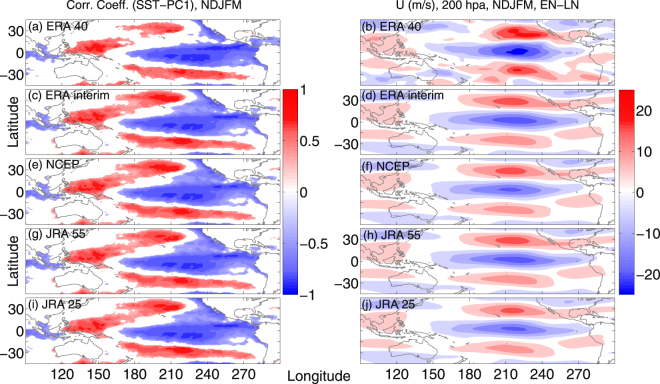
Correlation analysis between SST and EOF1 during EN and LN. (**a**,**c**,**e**,**g**,**i**) Represent significant (>95%) correlation coefficient between NDJFM mean PC1 and Hadley SST anomaly events for ERA 40 (1979–2002), ERA interim (1979–2012), NCEP (1979–2012), JRA 55 (1979–2012) and JRA 25 (1979–2012), respectively. (**b**,**d**,**f**,**h**,**j**) Represent the difference in 200 hpa NDJFM mean zonal wind (m/s) between EN and LN years for the respective datasets mentioned above. The maps in the figure are generated using the MATLAB software (Version: R2012b (8.0.0.783) & http://www.mathworks.com/products/matlab/?s_tid=srchtitle).

To analyze the spatial pattern in UT zonal wind, we conducted an EOF analysis of the winter mean zonal wind anomaly at 200 hPa in the spatial domain between 120°–280°E and 40°S–40°N. The principle component 1 (PC1) is the dominant one, which explains 45.4%, 54.2%, 44.6%, 46.7% and 51.2% of the total variance of the UT zonal wind for ERA 40, ERA interim, NCEP, JRA 55 and JRA 25, respectively (Supplementary Figure 1). The detrended PC1 exhibits significant trends in all the five datasets (not shown here).To illustrate the linkage between UT zonal wind and SST, we performed a correlation analysis between PC1 and winter mean (NDJFM) SST anomaly. In all five datasets, the correlation coefficient is very high (exceeding at least the 99% significance level) and even near unity. The correlation pattern is characterized by strong negative values centered over the equatorial central to eastern Pacific and a positive correlation region over the western Pacific, stretching eastward on either side of the equator (Fig. 2(a,c,e,g and i)). For the 200 hPa zonal wind field, the composite difference between EN (10 cases) and LN (12 cases) conditions exhibit stronger subtropical jets (either side of equator), which are stretching between 150°–280°E and 170°–250°E, respectively (Fig. 2(b,d,f,h and j)) and weaker equatorial westerlies in the EN cases.

**Figure 3:**
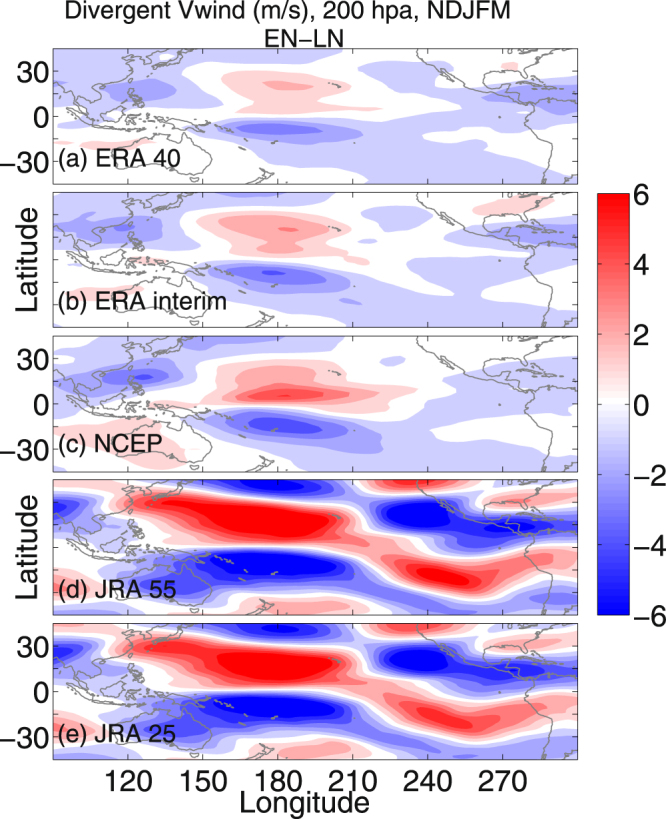
UT divergent wind during EN and LN. (**a**,**c**,**e**,**g**,**i**) Represent the difference (EN-LN) in 200 hpa NDJFM mean divergent wind (m/s, meridional component) for ERA 40 (1979–2002), ERA interim (1979–2012), NCEP (1979–2012), JRA 55 (1979–2012) and JRA 25 (1979–2012), respectively. The maps in the figure are generated using the MATLAB software (Version: R2012b (8.0.0.783) & http://www.mathworks.com/products/matlab/?s_tid=srchtitle).

One way to characterize the Pacific Walker circulation is the difference in sea surface pressure and temperature over Indonesia and the eastern tropical Pacific Ocean. The interannual variability is closely related to ENSO^32–34^. Normally, in the LN conditions a warm and wet tropical western Pacific (warm SST and low SLP) and cool and dry eastern Pacific (cold SST and high SLP) causes the surface air to move from east to west (Supplementary Figure 2(c) and (d)). Higher up in the atmosphere, west to east winds complete the circulation, forming westerly ducts over the equatorial Pacific Ocean^32,35^. The situation is just opposite in the EN conditions (Supplementary Figure 2(a) and (b)), resulting in a weakening of the Pacific Walker circulation and the equatorial westerly duct at 200 hPa. On the other hand, the Pacific Hadley circulations have two cells, with warm and moist air rising from the inter-tropical convergence zone (ITCZ), and then diverging northward and southward in the upper troposphere, finally descending over the subtropical highs^36^. Theoretically, air over the equator reaches 30° of latitude with an eastward component due to the Coriolis force. The upper branches of the Hadley circulation at 200 hPa conserve the angular momentum as they spiral inward toward the Earth’s axis of rotation, dumping their angular momentum into the subtropical jet stream. Figure 3(a,b,c,d and e) exhibit the composite difference in winter mean divergent wind (meridional component) between the EN and the LN years for ERA 40, ERA interim, NCEP, JRA 55 and JRA 25, respectively. In all five datasets, a strengthening of the divergent wind speed is prominent, which in turn strengthens the subtropical jet (Fig. 2, right panels) over the central Pacific (150°–210°E) under EN conditions.

### PV intrusion and its impact on Ozone concentration in two phases of ENSO

As mentioned before, PVIs influence the distribution of tracer constituents in the UT by mixing the ozone of stratospheric origin into the tropical UT^6^. Previous case studies show that the intrusions have weak signals in total columnar ozone, except during January 1987, when the intruded tongue extended deep downward to the mid troposphere^6^. However, these events act as an important source for the downward and equatorward transport of stratospheric air^28^ to the outer tropical (10°–25°N) troposphere^8^. Zonal mean stratospheric circulation, driven by ENSO and Quasi-Biennial Oscillation, has a significant impact on the tropospheric ozone levels^16^. Enhanced poleward and downward transport to the mid-latitudes happens during EN/easterly shear QBO and weakened transport is found for LN/westerly shear QBO. Furthermore, Neu *et al*.^14^ relate the decrease in UT ozone over the central-eastern Pacific Ocean to the enhanced convection under EN conditions and the increase of UT ozone to suppressed convection during the LN phase.

**Figure 4:**
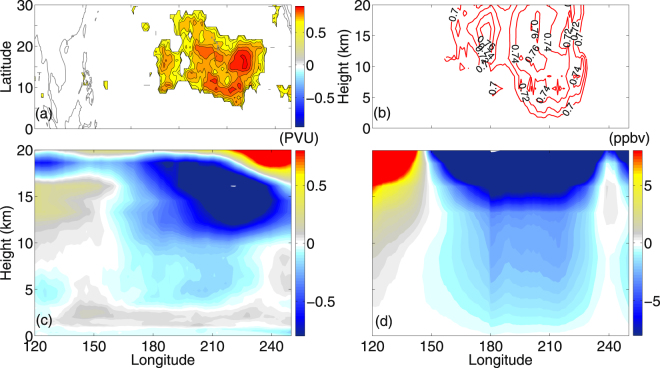
PV intrusion frequency and Ozone transport during EN and LN. (**a**) Latitude-longitude section of NDJFM mean (ERA40, ERA interim, NCEP, JRA55 & JRA25) correlation coefficient between PV (200 hpa) and TOMS total columnar ozone density 1983–2012. (**b**) Represents the longitude-height (10–25°N) section of mean (ERA40, ERA interim, NCEP, JRA55 & JRA25) correlation coefficient between intrusion frequency and AIRS ozone density. (**c**) Represents the difference in ERA interim PV (PVU, 200 hpa) between El-Nino and La-Nina years and (**d**) represents the same but for AIRS ozone density (ppbv). The maps in the figure are generated using the MATLAB software (Version: R2012b (8.0.0.783) & http://www.mathworks.com/products/matlab/?s_tid=srchtitle).

**Figure 5:**
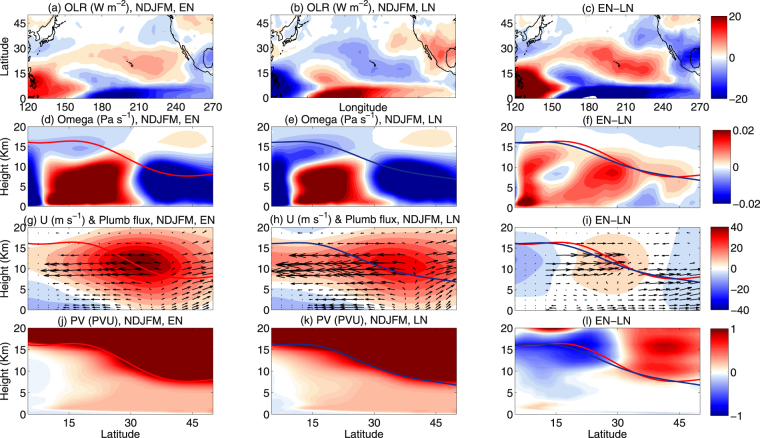
OLR (NOAA), vertical velocity, Plumb wave activity fluxes, tropopause height and PV (ERA interim) during EN and LN. (**a**,**b**,**c**) Represents latitude-longitude section of NDJFM mean NOAA interpolated OLR (W/m^2^) for EN, LN and the difference between EN & LN years, respectively. (**d**,**e**,**f**) Represent the latitude-height section of NDJFM mean of pressure vertical velocity i.e. Omega (Pa s^−1^) for EN, LN and the difference between EN & LN years, respectively and mean over 160–240°E. (**g**,**h**,**i**) Represent same as (**d**,**e**,**f**), but for U (color shading, m s^−1^) and Plumb wave activity fluxes (arrows, m^2^ s^−2^) and mean over 160–240°E. The PV (2PVU) tropopause heights are plotted in red and blue lines for EN and LN, respectively. (**j**,**k**,**l**) Represent same as (**d**,**e**,**f**), but for PV and mean over 160–240°E. The maps in the figure are generated using the MATLAB software (Version: R2012b (8.0.0.783) & http://www.mathworks.com/products/matlab/?s_tid=srchtitle).

In the present manuscript, we investigate the impact of PVI events on downward and equatorward transport of ozone in the two phases of ENSO. We found a strong correlation between PV (200 hPa) and TOMS total columnar ozone in the northern subtropical central-eastern Pacific (Fig. 4(a)). To ascertain the linkage, the correlation analysis is performed between Pacific PVI events (separately for ERA interim, NCEP, JRA 25, JRA 55) and AIRS ozone density (mean over 10–25°N), at individual pressure levels, right from the ground to 20 km height. Figure 4(b) exhibits the longitude-height section of the (mean of 4 datasets) significant correlation coefficients (all >99% significance level). In the inner tropical UT (0–10°N), the ozone anomalies are primarily due to convection associated with ENSO. It can be confirmed from the significant (>99%) correlation between Omega (500 hpa, 0–10°N) and ozone concentration is very high (0.8–0.85, not shown here), but restricted within the inner tropics only. Stronger upwelling is bringing up more and weaker upwelling is bringing up less ozone from the inner tropical UT. However, the situation is different in the subtropics. The factors influencing the ozone concentration and transport in the outer tropics are illustrated below in section (d). More details are available in paragraph 2 of section (d). Therefore, in consistency with the number of intrusion events, it is expected to have more ozone transport during the LN conditions and less under the EN conditions. To attribute these processes, we plotted the longitude-height section (mean over 10–25°N) of the composite difference of winter mean PV (from ERA interim) and ozone density (from AIRS) in Fig. 4(c and d), respectively. As expected, in the LS, PV is stronger in the LN phase, associated with enhanced downward penetration of ozone rich dry air into the LT, even to the marine boundary layer, over the northern subtropical central Pacific (160°–240°E). Within this region, the relative strength of the subtropical jet is weaker and the equatorial westerlies are stronger under LN conditions (see Fig. 5). This provides a favorable background for the intrusion of high PV air into the tropics. Based on the existing theory, baroclinicity of the mid-latitudes allows high PV air to penetrate into the LT and to interact with the surface fronts, whereas the subtropical intrusions are fairly barotropic and generally are restricted to the UT. Our present analysis illustrates the role of Rossby wave breaking events on the middle to lower tropospheric ozone concentration. Particularly the subtropical northern central Pacific under LN conditions is affected by this process (more details in Section (d)).

### Other factors affecting the ozone transport in the troposphere

Apart from the subtropical intrusions other factors controlling the tropospheric ozone trend need to be addressed here. E.g. tropical convection, mean vertical velocity and tropopause height in the two phases of ENSO. In the inner tropical UT, interannual ozone variability is mainly associated to the ENSO induced convective activity. Zonal mean stratospheric overturning circulation organizes the transport of ozone rich air poleward and downward to the high and midlatitudes leading there to higher ozone concentration^16^. In addition to this well described mechanism, an increase in ozone flux has been reported over the northern hemispheric outer tropical (10–25°N) central Pacific Ocean. This results from the equatorward transport and downward mixing from the midlatitude UT-LS to the outer tropics UT-LT during PV intrusions^8^.

To illustrate the convective activity over the Pacific Ocean in the two phases of ENSO, we plotted the NDJFM mean OLR anomaly during EN, LN and the difference between the two in Fig. 5(a,b and c), respectively. Under EN conditions, the convective activity is stronger in the equatorial and weaker in the outer tropical Pacific Ocean, which may strengthen the stratosphere-troposphere exchange (STE) and increase the ozone concentration in the stratosphere. The situation is opposite during LN conditions.

Hatsushika and Yamazaki^37^ showed that during EN the mean vertical velocity below 120 hPa becomes more positive over the eastern Pacific Ocean, which may work against mixing of ozone of stratospheric origin down into the troposphere. Therefore, to attribute the role of mean vertical velocity on ozone transport, we plotted the NDJFM mean pressure vertical velocity (omega) during EN, LN, and the difference between the two in Fig. 5(d,e and f), respectively. During EN condition, the central-eastern Pacific Hadley circulation is stronger, due to stronger upwelling over the equator (0–5°N) and downwelling over the subtropics (10–30°N). It favors stronger poleward and downward transport of ozone to the midlatitude UT. However, the stronger ascent of air parcels between 30–50°N inhibits the mixing of ozone downward below the tropopause (schematic diagram Fig. 1 of Neu *et al*.^14^). On the other hand, during LN, weakening of the central-eastern Pacific Hadley circulation weakens the stratospheric overturning circulation, which itself reduces the probability of mixing of stratospheric ozone down to the troposphere. Therefore, in the outer tropics (10–25°N) the possibility of mixing due to stratospheric overturning circulation is minimum (Fig. 1 of Neu *et al*.^14^) during LN. This is because the sinking branch of the stratospheric overturning circulation is somewhat away from the region of net increase in ozone density downward in the outer tropics, however, we cannot ruled out the possibility at all.

To illustrate the meridional transport of ozone fluxes due to PV intrusion or Rossby wave breaking events, we have plotted the NDJFM mean Plumb wave activity fluxes (mean over 160–240°E) in arrows during EN, LN, and the difference between the two in Fig. 5(g,h and i), respectively. To establish the causal linkage between the subtropical wave guide and the mean flow in different phases of ENSO, we have overplotted the zonal wind, mean over the season and longitudinal band mentioned above, during EN, LN, and the difference between the two in Fig. 5(g,h and i), respectively. During LN, the subtropical wave guide (7–17 km) is stronger and exhibits an equatorward and downward trajectory from the midlatitudes (UT–LS) to the outer tropics (UT–LT). This strengthening of the subtropical waveguide during LN is primarily due to the weakening of the subtropical jet (10–15 km & 30–40°N) and strengthening of the equatorial westerly duct (10–15 km), resulting from the weakening of the central-eastern Pacific Hadley circulation and Pacific Walker circulation, respectively^8^. The weakening of the regional Hadley circulation during LN is also evident from the difference plot of pressure vertical velocity in Fig. 5(f). Therefore, an increase in outer tropical central Pacific ozone density during LN appears to be linked strongly with the downward, equatorward and isentropic transport due to horizontal intrusions of midlatitude air.

Next, it is also essential to discuss the role of the tropopause height variability on STE processes in the different phases of ENSO. Hatsushika and Yamazaki^37^ showed that during the EN phase the tropopause height over the outer tropical central Pacific Ocean is higher compared to the LN phase. This suppresses downward mixing of ozone of stratospheric origin into the troposphere. In Fig. 5(g and h) we plotted the PV (2PVU) tropopause during EN and LN for the longitudinal band between 160°–240°E. From Fig. 5(i), the tropopause height appears to be higher by several 100’s of meters during EN in the outer tropical central Pacific Ocean. The region is collocated with the weakening center of the subtropical jet (~30°N and between 10–15 km) and the strengthening of the subtropical waveguide during the LN years. Although, the difference in tropopause height should have some impact on the transport and mixing of midlatitude air masses to the tropics, it is the strength of the UT westerly wind which acts as a wave guide for the Rossby waves to propagate deeper into the tropics.

**Figure 6:**
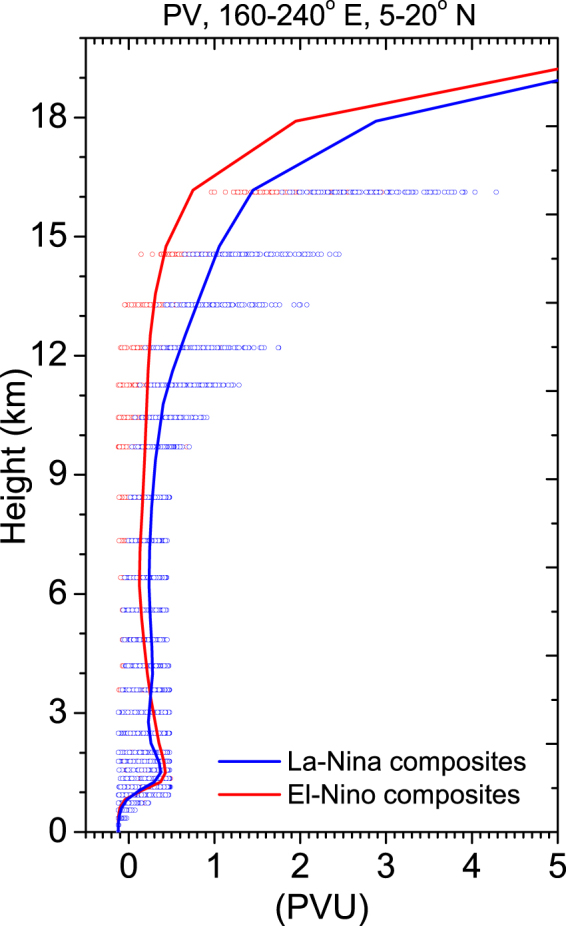
Vertical profile of PV during EN and LN. Scatter plots of PV between 5–20°N (mean over 160–240°E) from ERA interim as a function of height, during EN (red open circles) and LN (blue open circles) phases. The red and blue lines indicate the PV (PVU), mean over the region mentioned above, for EN and LN, respectively. It indicates the depth and strength of the intrusions.

It is argued that counting intrusion frequency is not necessarily the right metric for estimating its impact on tropospheric ozone. Several previous studies reported that it is the depth and strength of STE that matters, not the frequency^10,24^. Hsu *et al*.^15^ used a westerly duct event in 2001 to demonstrate that the intrusion signature in total ozone and in PV is large, but the real irreversible mixing is fairly small. In a recent study, using multiple datasets Nath *et al*.^8^ reported a long-term increase in PV intrusion frequency and ozone concentration in the outer tropics. To attribute the effect of strength and depth of PV intrusions, we plotted the latitude-height section (mean between 160° and 240°E) of NDJFM mean PV for the EN, LN and the difference between the two in Fig. 5(j,k and l), respectively. During the LN years, the magnitude of PV is less in the midlatitude LS, whereas, a narrow band of high PV air appears to propagate downward from the UT-LS to the LT over the outer tropical central Pacific (Fig. 5l), a region collocated with the observed increase in ozone density (Fig. 4d) and downward wave activity fluxes (Fig. 5i). Furthermore, Fig. 6 represents the scatter plot and mean of PV as a function of height, both for EN and the LN years. During the LN years, it clearly displays an increase in PV magnitude from the UT downward to the LT, at least to 3 km altitude.

**Figure 7:**
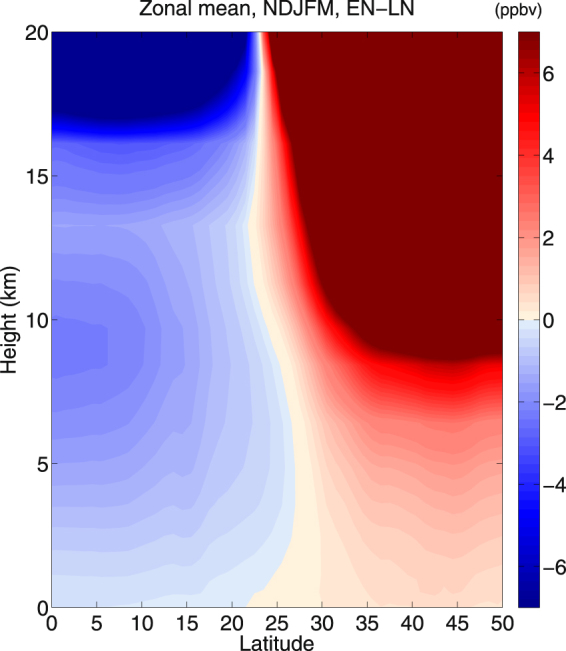
Zonal mean ozone abundances. Latitude-height section of differences in zonal mean ozone abundances within 10–25°N, between EN and LN years.

Therefore, from the above discussion we can conclude that tropical convection, stratospheric overturning circulation, vertical velocity and variability of tropopause height should have some impact on ozone transport and mixing in the outer tropical central Pacific Ocean. However, the subtropical PV intrusions due to Rossby wave breaking and ozone concentration displays at least 61–64% of the co-variability over that region^8^. In LN years, not only the intrusion frequency is enhanced, but the intrusion depth and strength of PV increase as well, facilitating stronger equatorward and downward transport of midlatitude air to the outer tropical central Pacific. However, it is worth mentioning in this context that covariance does not establish any causal linkage between the two. Therefore, to supplement the covariability we propose a mechanism which links the changes in UT zonal wind and intrusion of ozone rich air from midlatitudes to the tropics during LN years. This strengthens the STE processes and may increase the tropospheric ozone concentration between 10–25°N. More details on the mechanisms are stated in Nath *et al*.^8^. Additionally, intrusion frequency might be related to ozone transport but itself is not a transport quantity. Therefore, we perform height wise correlation coefficient between ozone concentration and PV, which displays a significant covariance of ~30–50% between 12 and 3 km (Supplementary Figure 3). Furthermore, to confirm the significance of the proposed mechanism we checked the sign of zonal mean ozone abundances in lower troposphere over the latitude band 10–25 N in the EN minus LN composite (Fig. 7). The ozone abundance is strongly negative throughout the lower troposphere, indicating the relative importance of intrusion events through the westerly duct in determining the outer tropical lower tropospheric ozone abundances. However, the mechanism proposed by Neu *et al*.^14^ i.e. stronger BDC, higher ozone concentration in LS and more ozone transported to the outer tropics through the subtropical jet region during EN phase is playing secondary role here.

### Interdecadal variability of PV intrusion frequency and shift of Pacific SST patterns

The variability of sea surface temperature (SST) over the tropical Pacific basin involves multiple time scales, and affects the atmospheric and oceanic circulation both in local and remote scales. Since the end of the last century, the tropical Pacific Ocean is undergoing rapid dynamic changes and experienced a climate regime shift (CRS ~1996/97) visible by the changed Pacific SST pattern^9^. This decadal SST shift is characterized by a warming over the equatorial western Pacific, the mid-latitude North and South Pacific, and cooling in the equatorial central Pacific. This CRS in the Pacific Ocean is induced by a shift towards the LN like state (at least until the onset of the 2015/16 El Niño) with cold SST in the eastern tropical Pacific driven by a strengthening of trade winds in the Pacific Ocean^36^ during the negative phase of the Interdecadal Pacific Oscillation (IPO). The recent inter-decadal abrupt warming of the western Pacific was identified to have occurred around late 1998/1999^38,39^. It is also argued that, in the recent decade, the Central Pacific Ocean acts as a key pacemaker for the variable rates of global warming^38^ and climatic changes both in local and remote scale. Furthermore, this CRS is the cause of El Niño behavior change from a dominant eastern Pacific warming type to a dominant central Pacific warming type^39,40^. Therefore, the interdecadal variability in the Pacific Ocean dynamics, before and after 1998 should have significant impact on the UT zonal wind, PV intrusion frequency, and ozone transport in the outer tropics.

**Figure 8:**
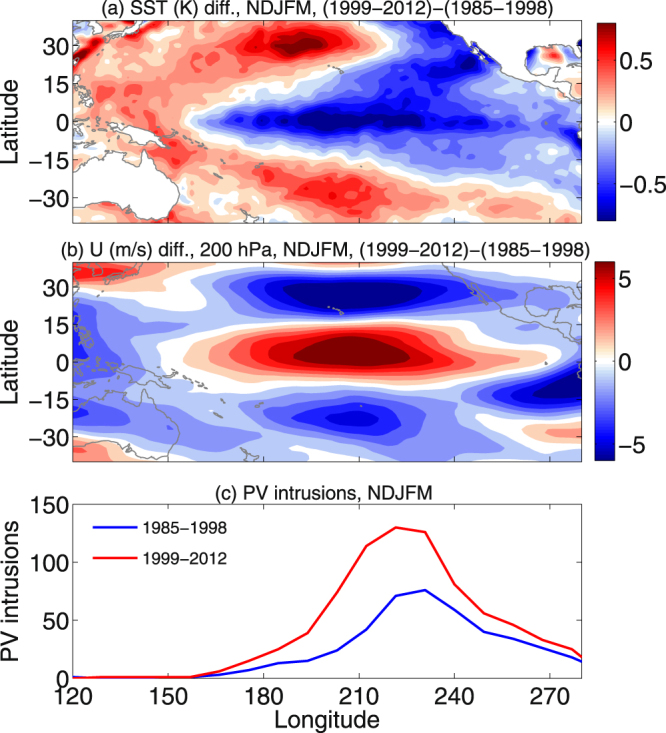
Interdecadal variability of SST, UT zonal wind, PV intrusion and Ozone transport. (**a**) and (**b**) represent NDJFM mean difference between 1999–2012 and 1985–1998 for SST (K) and U (m s^−1^, 200 hPa), respectively. (**c**) Displays PV intrusion frequencies during 1985–1998 (blue) and 1999–2012 (red), as a function of longitude and sum over NDJFM. The maps in the figure are generated using the MATLAB software (Version: R2012b (8.0.0.783) & URL: http://www.mathworks.com/products/matlab/?s_tid=srchtitle).

Nath *et al*.^8^ showed that the variability in UT zonal wind is tied closely with the changes in SST anomalies, particularly in the Pacific Ocean. They observed a long-term increasing trend in PV intrusion frequency since 1979. To illustrate the interdecadal variability over the Pacific Ocean, we plotted the difference in SST, zonal wind at UT (200 hPa) and PV intrusion statistics between 1999–2012 and 1985–1998 in Fig. 8(a,b and c), respectively. The difference in SST exhibits the conventional LN like condition in the tropical Pacific Ocean, with horse-shoe shaped warming pattern in the western Pacific and a cooling pattern in the tropical eastern Pacific Ocean. Furthermore, strengthening of the equatorial westerly winds and weakening of the STJ over the central Pacific is evident in the UT (Fig. 8b). The mechanism was illustrated in Nath *et al*.^8^. The zonal gradient in SST due to the strong LN like condition over the Pacific Ocean from 1998 onwards drives enhanced convective activity in the western Pacific leading to enhanced precipitation. The latent heat released in the process strengthens the Pacific Walker circulation and the westerly duct over the equator. On the other hand, the cooling trend over the central to eastern Pacific and related sinking air suppress convective activity. This process after 1998 weakens the central-eastern Pacific Hadley circulation and the subtropical jet by weakening of the poleward divergent flow in the UT. Since the changes in SST anomalies, UT zonal wind and the PV intrusions are interrelated, it is essential to investigate the difference in intrusion characteristics and ozone transport due to Rossby wave breakings before and after 1998. The composite of all the intrusion events during 1985–1998 and 1999–2012 (Fig. 8c) displays a two-fold increase of PV intrusion events towards the later decade. Not only the intrusion frequency is enhanced, also the distribution of the events widens zonally and the frequency peak shifts gradually westward toward the central Pacific Ocean (from 230°E to 220°E). This should have a significant impact on the interdecadal variability, transport and mixing of ozone, water vapor and other trace constituents in the outer tropical central Pacific Ocean. Therefore, we may argue that the interannual oceanic and atmospheric variability over the Pacific Ocean itself is being altered by the recent CRS around 1997/1998 and that a LN like condition dominates the trends.

## Conclusions

**Figure 9:**
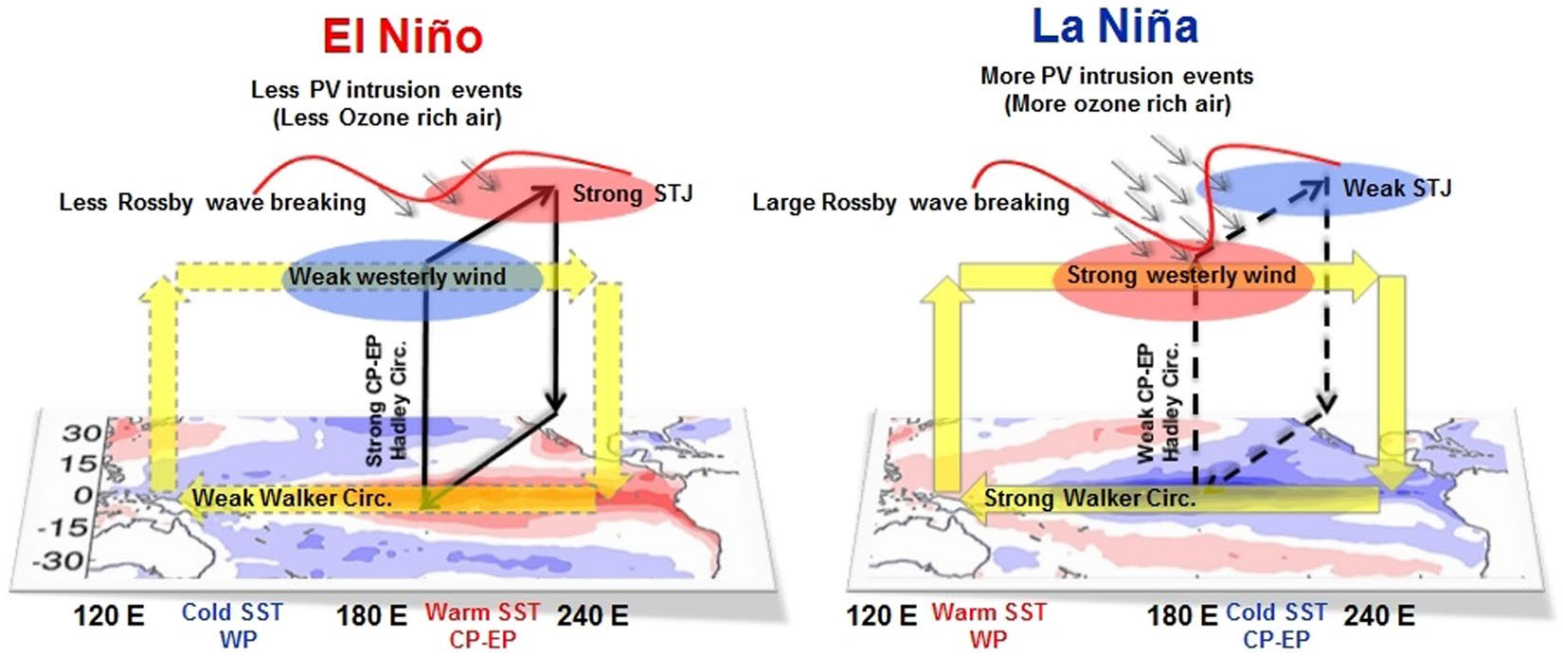
Schematic diagram during EN and LN conditions. It represents the differences in SST, tropical circulations and PV intrusions (200 hPa) during the El-Niño (left panel) and La-Niña (right panel) conditions. Bold and dashed arrow (black) indicates the strong and weak Central-Eastern Pacific Hadley circulation, respectively. Bold and dashed yellow arrows indicate the strong and weak Pacific Walker circulation, respectively. The maps in the figure are generated using the MATLAB software (Version: R2012b (8.0.0.783) & URL: http://www.mathworks.com/products/matlab/?s_tid=srchtitle).

We examined the climatology of PV intrusions and its impact on the tropospheric ozone distribution over the outer tropical Pacific Ocean during the two phases of ENSO. In all five reanalysis datasets the intrusion frequency is much higher during the cold LN phase, in comparison to the warm EN phase of ENSO. The climatological patterns of the UT zonal wind, PV and divergent wind show significant differences between the LN and EN conditions, which lead to differences in the Rossby wave characteristics. During LN, the UT equatorial westerly wind is stronger due to the strengthened Pacific Walker circulation, leading to more Rossby wave breaking and intrusions of midlatitude air to the tropics. In contrast, the situation is just opposite during EN. On the other hand, the divergent wind speed is stronger over the central Pacific under EN conditions, which in turn strengthens the subtropical jet (over the central to eastern Pacific Ocean) in boreal winter and spring months. A schematic diagram representing the differences in SST, tropical circulation and PV intrusions during EN and LN is shown in the left and right panels of Fig. 9, respectively.

The subtropical intrusions can extend from the midlatitude UT-LS to the tropical mid troposphere, but the downward penetration to the LT is restricted to a small percentage of cases. These PV intrusions play an important role in determining the chemical composition, particularly ozone, in the tropical troposphere. Our analysis of AIRS O3 measurements over the central-eastern Pacific Ocean displays large variability in O_3_ concentration, and much of these variations are associated with intrusion events, particularly under LN conditions. In the middle–lower troposphere, the correlation coefficient between ozone density and PVI events is very high (~0.8), which indicates that PVI join ~65% of the interannual variability of tropospheric ozone in the northern subtropical central Pacific Ocean. While this does not establish a causal link, it shows the very strong statistical linkage between PVI and tropospheric ozone in the tropics. We suggest a mechanism linking the changes in tropical circulations to the UT zonal wind, PV intrusions and ozone transport over Pacific Ocean in the two phases of ENSO. The relative importance of the proposed mechanism is evident from the negative ozone concentration, in determining the lower tropospheric ozone abundances in the outer tropical Pacific Ocean. The role of stronger BDC in ozone transport during the EN years plays secondary role here. We also highlight the contribution of other factors, e.g. stratospheric overturning circulation, tropical convection, vertical velocity and tropopause height variability, which may drive ozone transport and mixing in the outer tropics. Furthermore, we observe that not only the frequency of the intrusions, but also the strength and depth of PVI increases poleward and downward to the LT, during LN.

Apart from the interannual variability, the CRS in the Pacific SST pattern during 1997/98 drives the interdecadal variability in the UT wind pattern. It strengthens and weakens the equatorial westerly wind and the subtropical jet, which may be resulting from anomalous warming over the western Pacific warm pool and cooling of the tropical eastern Pacific. At the same time, a two-fold increase in intrusion frequency can be seen from 1985–1998 to 1999–2012. Not only the intrusion frequency has changed, but also the distribution of the events widens zonally and the frequency peak shifts gradually westward (from 230°E to 220°E) toward the central Pacific Ocean.

Despite the fact that our present analysis confirmed a close linkage between the subtropical PV intrusions and middle-lower tropospheric O_3_ distribution, particularly in the LN phase, it did not address the amount of irreversible transport of O_3_ and its interaction with surface fronts in the Pacific marine boundary layer. Along with ENSO, the role of QBO on PVI statistics and ozone distribution needs to be examined in more detail. Moreover, the impact of PVI events on other trace constituents, e.g. water vapor, is an important issue to pursue. Furthermore, in the present manuscript we show the covariability between subtropical intrusions and tropospheric ozone concentration and propose the mechanism. However, a proper ozone budget study is essential using the TEM continuity equations and quantifying the residual advection and eddy transport from the model simulated datasets^41–43^.

## Electronic supplementary material

Supplementary Information
