# Supplementary material for: Contrasting subtropical PV intrusion frequency and their impact on tropospheric Ozone distribution over Pacific Ocean in El-Niño and La-Niña conditions
Source: Sci Rep. 2017 Sep 20;7:11987. doi: 10.1038/s41598-017-12278-7 (PMC5607222; doi:10.1038/s41598-017-12278-7)
Supplement: Supplementary file 1 — Supplementary Information [file 41598_2017_12278_MOESM1_ESM.pdf]

# Contrasting subtropical PV intrusion frequency and their impact on tropospheric Ozone distribution over Pacific Ocean in El-Niño and La-Niña conditions

Debashis Nath[1*], Wen Chen[1*], Hans-F. Graf[2], Xiaoqiang Lan[1], Hainan Gong[1]

[1]*Center for Monsoon System Research, Institute of Atmospheric Physics, Chinese Academy of Sciences, Beijing 100190, China*

[2]*Center for Atmospheric Science, University of Cambridge, Cambridge, UK*

***Corresponding Author**

1. Wen Chen, Professor/Director

Center for Monsoon System Research,

Institute of Atmospheric Physics, CAS

Beijing: 100029, China

Ph: +86 13681102073, Email: cw@post.iap.ac.cn

2. Debashis Nath, Professor (Associate)

Center for Monsoon System Research,

Institute of Atmospheric Physics, CAS

Beijing: 100029, China

Ph: +86 18310814995, Email: debashis@mail.iap.ac.cn

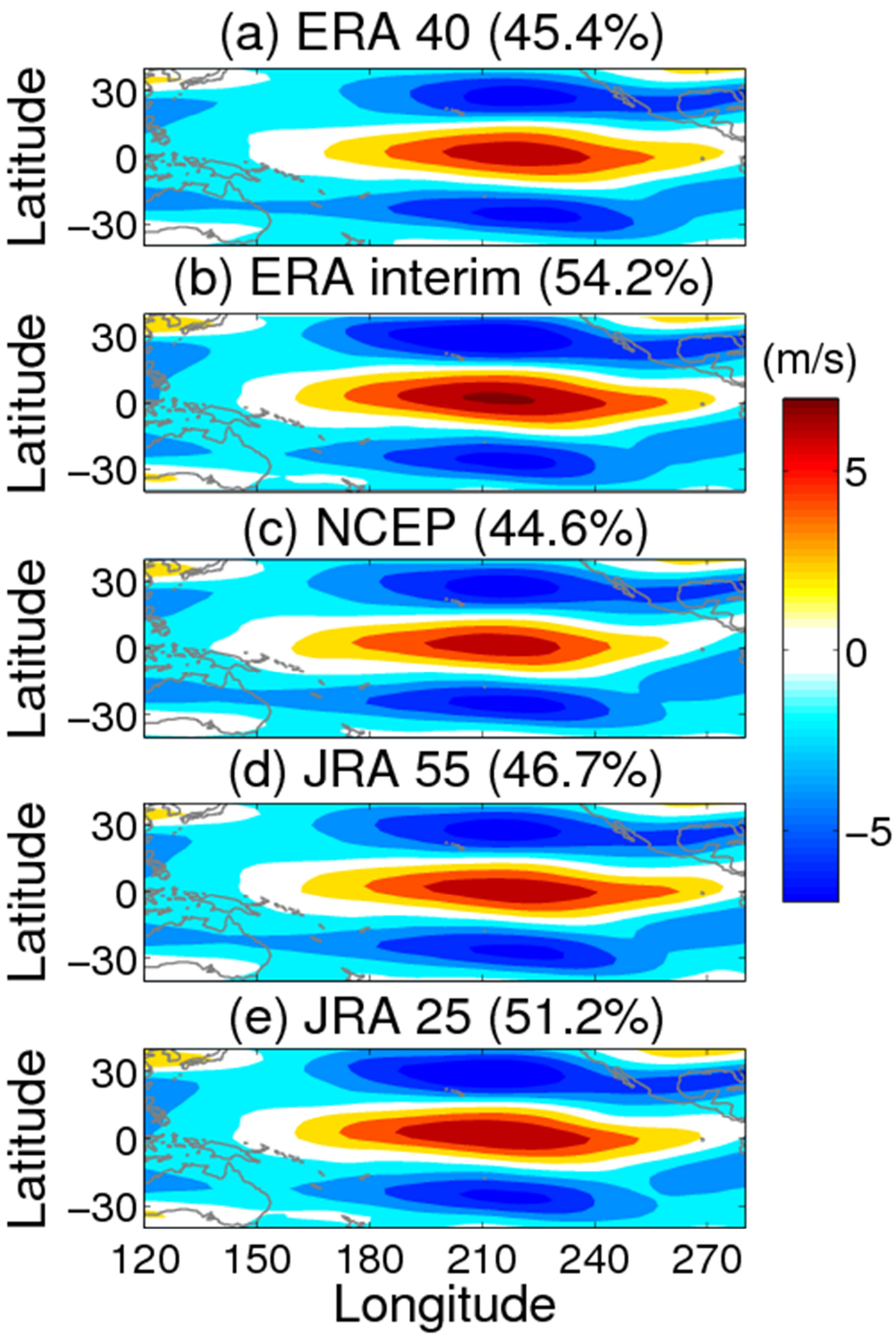


**Supplementary figure 1.** Spatial EOF1 (120°–280°E, 40°S–40°N) regression pattern of UT zonal wind (DJFM) for (a) ERA40, (b) ERA interim, (c) NCEP, (d) JRA55, and (e) JRA 25. The labeled percentages indicate the percentage of the total variance explained by PC1, the most dominant one. The maps in the figure are generated using the **MATLAB** software (Version: R2012b (8.0.0.783) & URL: http://www.mathworks.com/products/matlab/?s_tid = srchtitle).

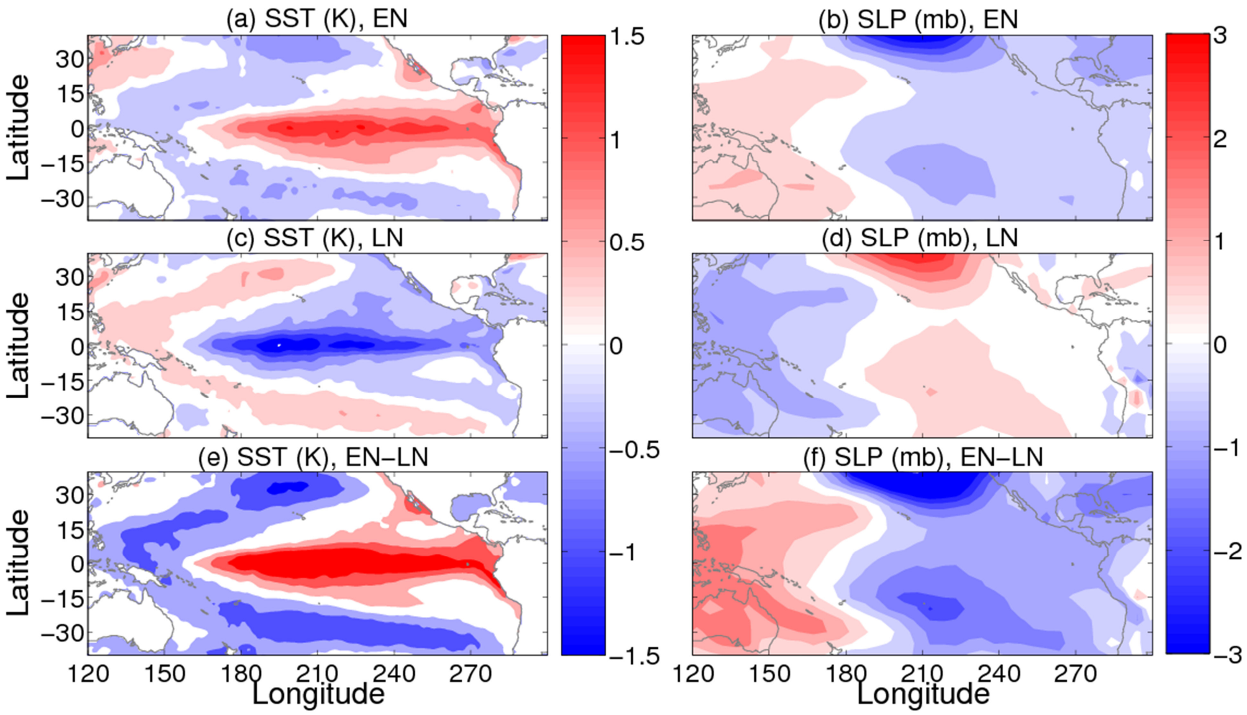


**Supplementary figure 2.** (a) and (b) represent the NDJFM mean Hadley SST (K) and SLP (mb) anomaly for the composite El-Nino (EN) years, respectively. (c) and (d) & (e) and (f) represent the same but for the composite La-Nina (LN) years and difference between EN and LN years, respectively. The maps in the figure are generated using the **MATLAB** software (Version: R2012b (8.0.0.783) & URL: http://www.mathworks.com/products/matlab/?s_tid = srchtitle).

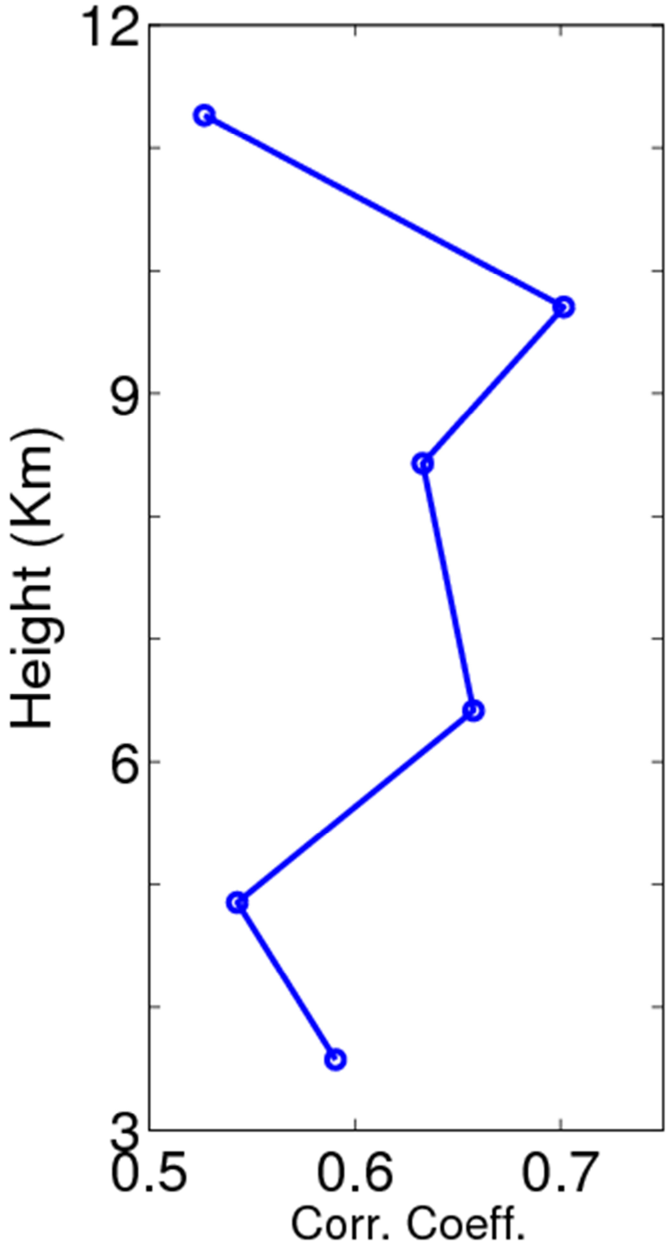


**Supplementary figure 3.** Height wise significant (>95%) correlation coefficient between PV and Ozone concentration from AIRS.
